# Fournier’s gangrene after adult male circumcision

**DOI:** 10.1186/s12245-014-0037-0

**Published:** 2014-09-24

**Authors:** Moses Galukande, Dennis Bbaale Sekavuga, Alex Muganzi, Alex Coutinho

**Affiliations:** 1 International Hospital Kampala, Namuwongo P.O. Box 8177, Kampala, Uganda; 2 Infectious Diseases Institute, Makerere University, Kampala, Uganda

**Keywords:** Gangrene, Male, Adult, Circumcision

## Abstract

**Background:**

In the advent of mass voluntary medical male circumcision (VMMC) for the partial prevention of HIV, previously rare adverse events associated with adult male circumcision are likely to be encountered with higher frequency. Fournier’s gangrene, defined as a polymicrobial necrotizing fasciitis of the perineal, perianal or genital areas, is one such rare and life-threatening adverse event. In this report, we present two cases that were identified in the context of a VMMC programme over a 3-year period during which approximately 100,000 adult circumcisions were performed.

**Case presentations:**

Case 1: A 19-year-old male who had VMMC performed using the dorsal slit technique developed pain and blisters on the scrotal skin on the sixth postoperative day. He had no co-morbidities, and serology for HIV was negative. On examination, locally he had scrotal skin necrosis with an offensive odour and was dehydrated but afebrile. Repeated aggressive debridement was done while he stayed in a hospital for 3 weeks; at which point, he had healthy granulation tissue and was free of infection. The wound had closed spontaneously and completely by the fifth month.

Case 2: A 52-year-old male who had VMMC performed with the sleeve resection method developed pain and swelling of the penis and scrotum on the fourth postoperative day. He had a low-grade fever of 37.6°C. He was not diabetic or immunosuppressed and had a negative HIV serology. He was admitted and was given IV antibiotics, and repeated aggressive debridement was performed. On the third week of hospitalization, he had healthy granulation tissue and received a split skin graft on the penile shaft. At 4 months, the scrotal defect had completely closed.

**Conclusion:**

Fournier’s gangrene is a rare occurrence after adult male circumcision with associated high morbidity. These are the first descriptions in the VMMC era.

## 1 Background

In the advent of mass voluntary medical male circumcision (VMMC) for the partial prevention of HIV [1], previously rare adverse events associated with adult male circumcision will likely be encountered. Fournier’s gangrene is an infection, often polymicrobial in nature with necrotizing fasciitis involving the perineal and genital regions. It carries a high mortality rate and continues to be a major challenge to the medical community [2]. Fournier’s gangrene was first identified in 1883, when the French venereologist Jean Alfred Fournier described a series in which five previously healthy young men suffered from a rapidly progressive gangrene of the penis and scrotum without an apparent cause. In contrast to Fournier’s initial description, the disease is not limited to young people or to males, and a causative agent is now usually identified [3].

In this paper, we describe two cases of Fournier’s gangrene that occurred in two adult males after voluntary circumcisions were performed on them in Uganda.

## 2 Case presentations

The occurrence of these cases was over a 3-year period during which slightly over 100,000 VMMCs were performed. These circumcisions were performed by the Infectious Disease Institute and International Hospital Kampala (IHK) collaboration supported by Centers for Disease Control and Prevention (CDC), and the procedures took place in both rural and urban regions. These occurrences of Fournier’s gangrene were 2 weeks apart and occurred at two different sites with procedures performed by two different teams. The first site is a rural site in western Uganda, and the second site is a peri-urban area in central Uganda.

These case descriptions are events picked from an active daily adverse event (AE) reporting mechanism. The mechanism comprises of an initial 24-h report of client status and a follow-up of up to 6 weeks. On the seventh POD, a physical examination is done and phone call surveillance after 6 weeks. In addition, a hotline is available and accessed by the clients who needed to address any query they had.

### 2.1 Case report 1

**Figure 1:**
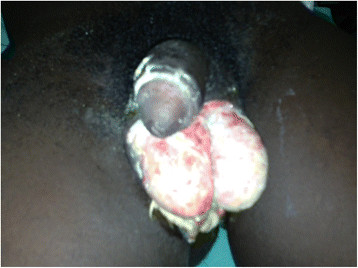
A picture of case report 1 on day 8.

**Figure 2:**
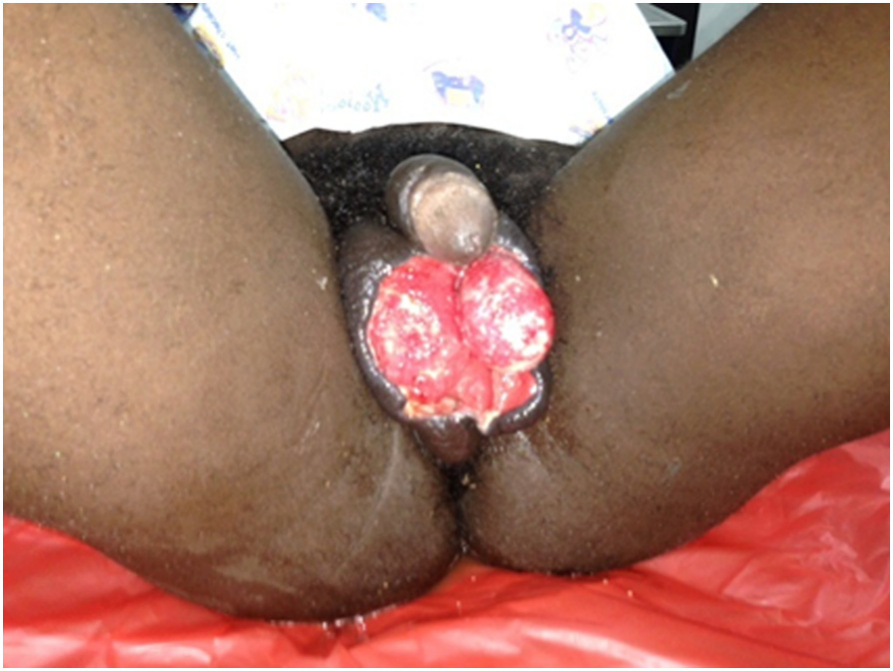
A picture of case report 1 on day 19.

**Figure 3:**
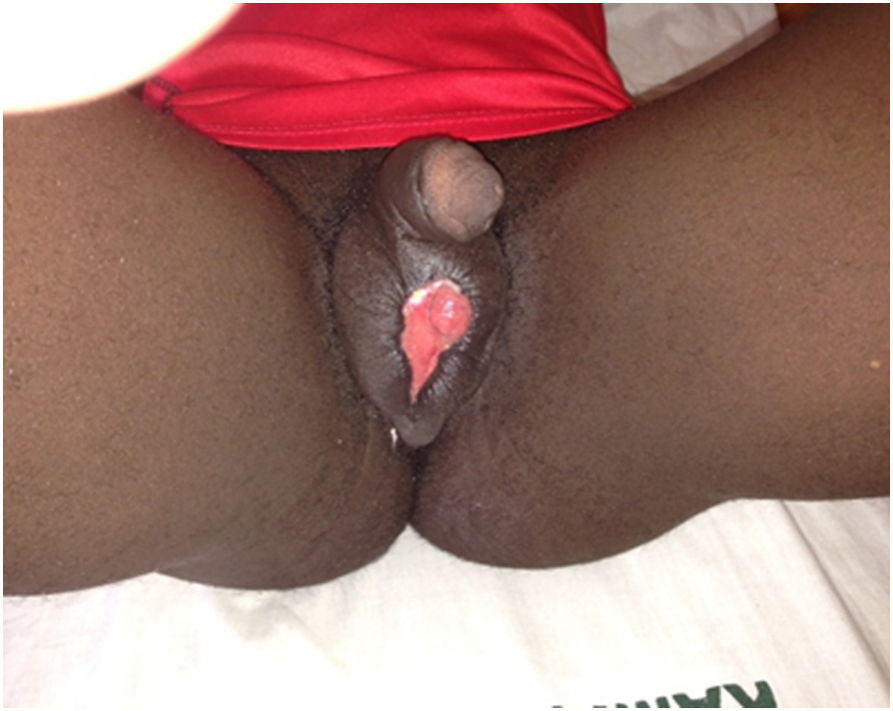
A picture of case report 1 on week 7.

**Figure 4:**
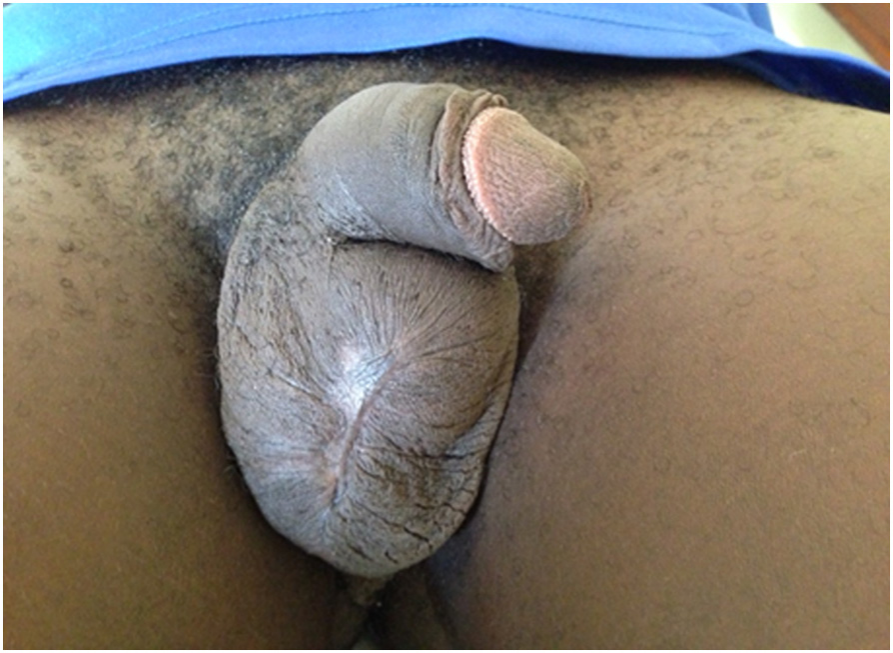
Shows a full healed wound after 5 months for case 1.

A 19-year-old male with no underlying illnesses developed pain and blisters on the scrotal skin 6 days after VMMC using the dorsal slit technique. On examination, he had scrotal skin necrosis with an offensive odour and was dehydrated but afebrile. On admission, he had a normal random blood sugar, Hb 11.2 g%, white cell count of 5,200 with 74% neutrophils and HIV serology was negative. Culture and sensitivity (C&S) results showed no bacterial growth/isolates. Though the patient had prior antibiotics on the way to the hospital, he was administered with intravenous fluid and broad spectrum antibiotics (ceftriaxone and metronidazole). An initial debridement was done to remove all devitalized tissues (see Figure 1), and subsequently repeated aggressive debridement was performed on the first week of admission. He developed an abscess over the left inguinal area that was incised with 500 cc of pus was drained over 4 days. At the end of the 3-week period in a hospital (see Figure 2), he had healthy granulation tissue and was free of infection. Wound care with dressing changes every 3 to 4 days (see Figure 3) were conducted until there was spontaneous wound closure 5 months later (see Figure 4).

### 2.2 Case report 2

**Figure 5:**
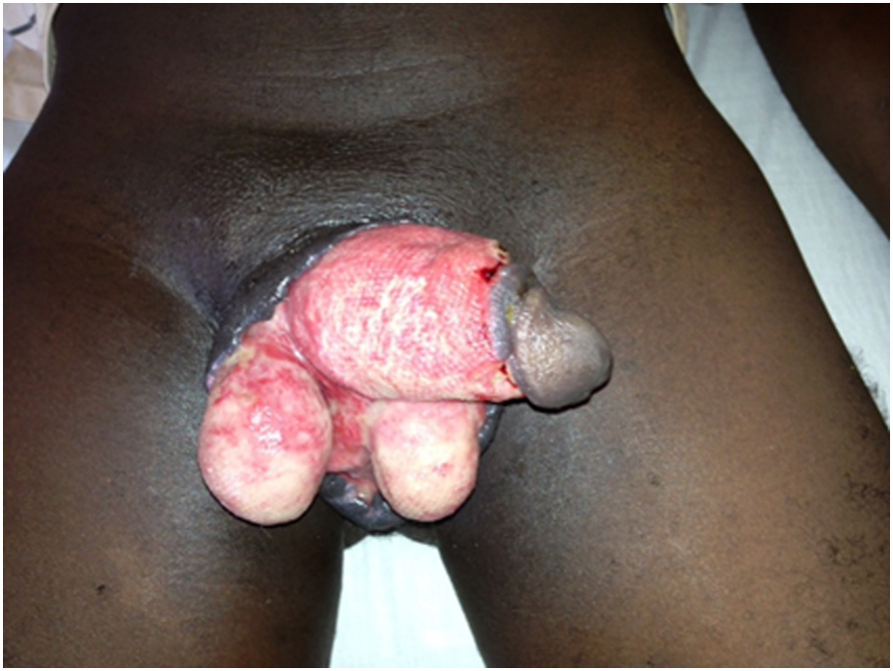
A picture of case report 2 on day 7.

**Figure 6:**
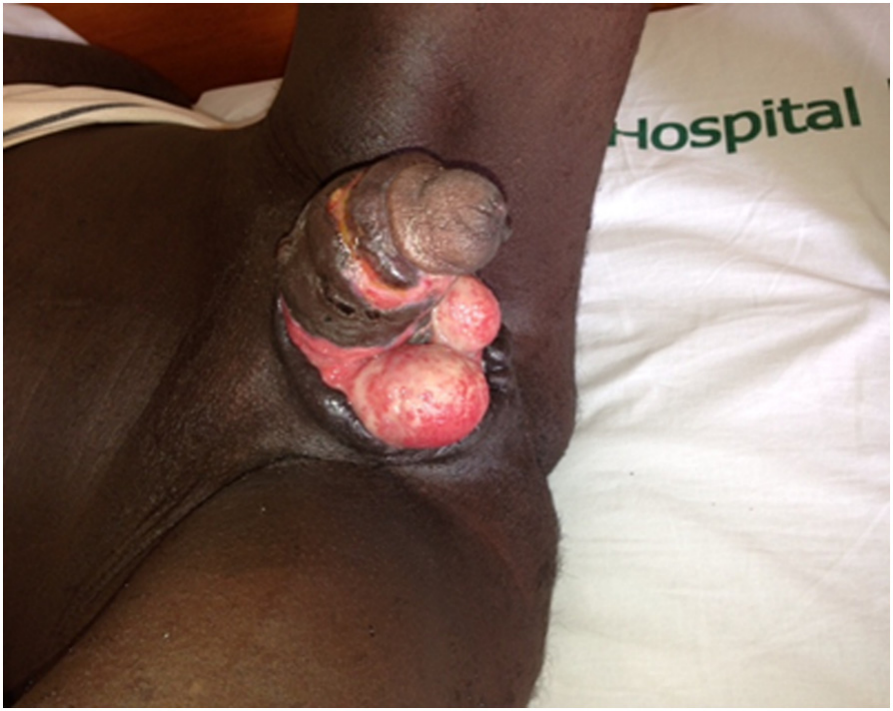
**A picture of case report 2.** Penile split skin graft a week after grafting and a month from occurrence of event.

**Figure 7:**
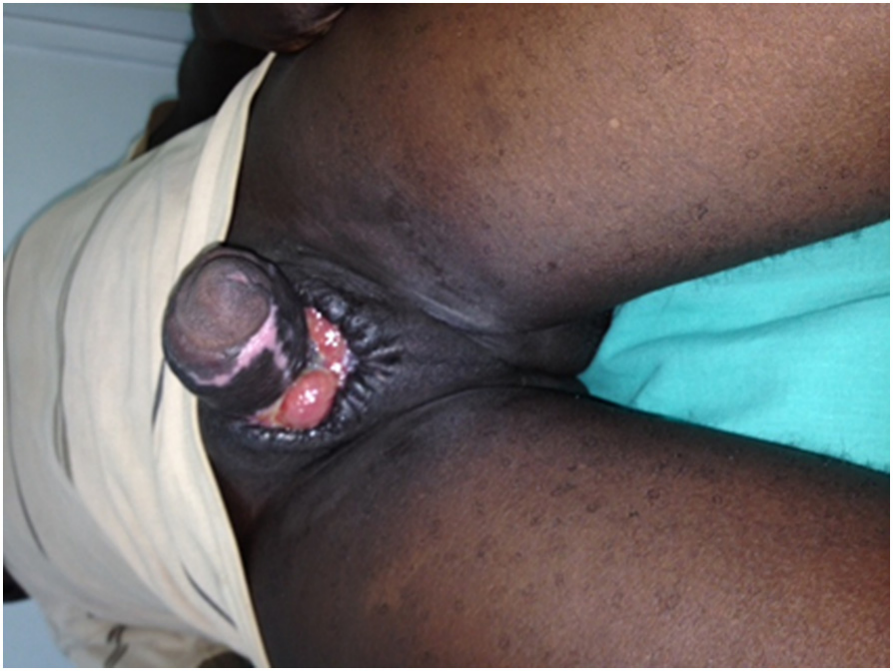
A picture of case report 2 after 3 months from time of event.

**Figure 8:**
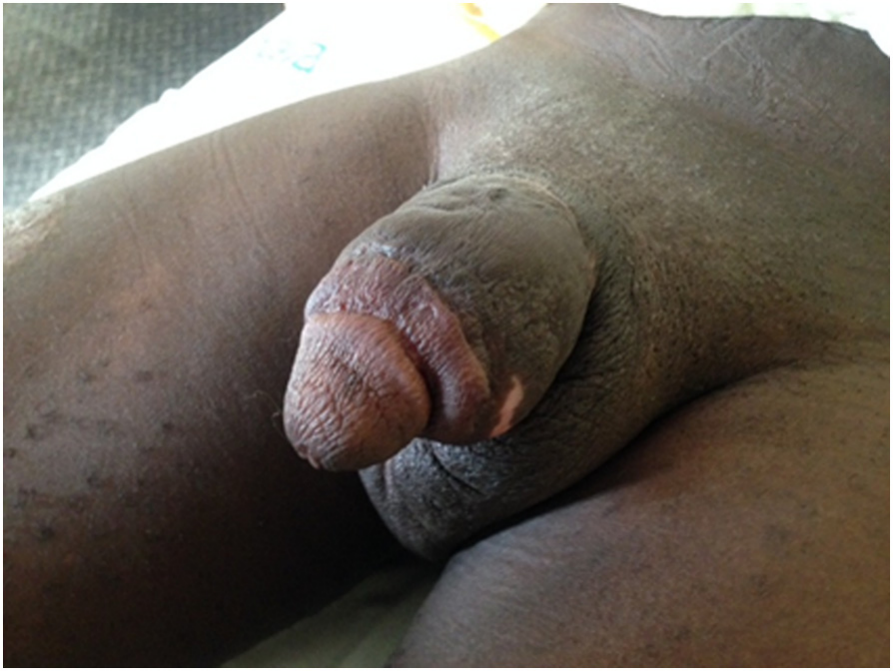
A picture of case report 2 when healed after 4 months.

A 52-year-old man developed painful swelling of the scrotum and penis 4 days after an uneventful surgical circumcision using the sleeve resection technique. There was no history of diabetes, hypertension or alcohol abuse. Fasting blood sugar at the time of presentation was within normal range, and HIV serology was negative. The white cell count was elevated at 12,000/mm3 with a neutrophil count of 78%; no growth was obtained from the cultures. He was admitted at a local clinic near his home for a week, he underwent aggressive debridement three times before the wound was free of necrotic tissue and developed a healthy granulation tissue (see Figure 5). He was transferred to a hospital where further evaluation was done, and he underwent a split skin graft (see Figure 6) onto the penile shaft that was previously devoid of skin cover. The scrotal defect was dressed every 3 to 4 days until healing was complete on the 5 month post event (see Figures 7 and 8).

A telephonic interview 10 months later revealed no sequel, and the patient reported resumption of normal sexual activity that had occurred 5 months earlier.

## 3 Discussion

Reports of Fournier’s gangrene after adult circumcision are lacking in the medical literature. In this programme, we report a 1 in 50,000 occurrence; however, with the advent of mass male circumcision in sub-Saharan Africa, we are likely to see more adverse events. Treatment of Fournier’s gangrene, regardless of etiology, involves aggressive debridement, intravenous antibiotics, fluid resuscitation and management of underlying morbidities such as diabetes, if present, by controlling the blood sugar [4],[5].

Impaired immunity (e.g. from diabetes) is a risk factor for Fournier’s gangrene; however neither of these patients were immunosuppressed, demonstrating that it may occur in normal hosts as well. Trauma to the genitalia is a frequently recognized vector for the introduction of bacteria that initiate the infectious process where obliterative endarteritis develops, and the ensuring cutaneous and subcutaneous vascular necrosis leads to localized ischemia and further bacterial proliferation [3]. In the cases described here, the circumcision wound allowed introduction of infection leading to necrotizing fasciitis. It is not clear whether these patients had unknown predisposing underlying immunosuppressive risk factors for Fournier’s gangrene. Although wound care instructions for all males undergoing circumcision are provided, it is not uncommon as gathered from community feedback that some patients use traditional practices such as applying ash and other substances to the wound in an attempt to accelerate healing.

With aggressive treatment, both of the two cases described here recovered. Case 2 required a split skin graft of the penile shaft as it was completely denuded of skin. In case 1, there was tracking of pus to the lower abdominal wall that manifested as an inguinal abscess. Infection of the superficial perineal fascia (Colles’ fascia) may spread to the penis and scrotum via Buck’s and Dartos’ fascia or to the anterior abdominal wall via Scarpa’s fascia or vice versa [6]. The scrotal defects required no re-epithelialization techniques or maneuvers; the exposed testes developed healthy granulation tissue and spontaneous re-epithelialization though complete healing took 4 to 6 months.

### 3.1 Study limitation

Not all clients come back for a day-7 physical examination therefore introducing the possibility of similar events not being reported. However, Fournier’s gangrene is such a dramatic event that whoever gets it is likely to come back to a facility for urgent care.

## 4 Conclusions

Fournier’s gangrene is a rare adverse event after adult male circumcision. Because it can be life-threatening, providers in VMMC programmes need to be familiar with the rapid identification and management of this serious condition. The two cases described here were identified shortly after symptom onset, were managed aggressively at a referral center and survived with no serious medium-term consequences. In cases when this condition is not recognized or treated, early serious consequences are possible such as amputation of genitalia or death.

## 5 Consent

Written informed consent was obtained from the clients including consent to publish this information and the use of images. The International Hospital Ethics committee approved of the study.

## Abbreviations

AE: Adverse events

C&S: Culture and sensitivity

POD: Postoperative day

## Competing interests

The authors declare that they have no competing interests.

## Authors’ contributions

GM performed the surgical debridement and wrote the first draft. BD, AM and AC performed critical reviews of the manuscript for intellectual content and participated in the clinical care of the patient. All authors read and approved the final manuscript.
